# Influence of Phosphorus Sources on the Compressive Strength and Microstructure of Ferronickel Slag-Based Magnesium Phosphate Cement

**DOI:** 10.3390/ma15051965

**Published:** 2022-03-07

**Authors:** Cuirong Yan, Hongyan Ma, Zhongqiu Luo, Xintao Zhou, Luxing Wang

**Affiliations:** 1 Faculty of Chemical Engineering, Kunming University of Science and Technology, Kunming 650500, China; huaxiarong@126.com (C.Y.); wlx1234562022@163.com (L.W.); 2 Faculty of Environmental and Chemical Engineering, Kunming Metallurgy College, Kunming 650033, China; 3 Department of Civil, Architectural and Environmental Engineering, Missouri University of Science and Technology, Rolla, MO 65401, USA; mahon@mst.edu

**Keywords:** magnesium phosphate cement, electric furnace ferronickel slag, phosphorus source, hydration product

## Abstract

Electric furnace ferronickel slag (EFS) is a typical magnesium-rich industrial by-product discharged from the manufacture of nickel and iron-nickel alloys. The approach to use it as the raw material for the preparation of magnesium phosphate cement (MPC) has potential and proves effective. In this study, three different phosphorus sources (PS) including phosphoric acid (H_3_PO_4_, PA), sodium dihydrogen phosphate (NaH_2_PO_4_, SDP) and potassium dihydrogen phosphate (KH_2_PO_4_, PDP) were used to react with EFS to prepare the EFS-based MPC (EMPC), and the effects of raw material mass ratio (EFS/PA, EFS/SDP, EFS/PDP) on the compressive strength, early hydration temperature and microstructure of EMPC pastes were investigated. Results showed that the compressive strength of EMPC paste is significantly impacted by the type of phosphorus source and the raw materials mass ratio. When the EFS/PDP ratio is 4.0, the compressive strength of the MPC paste reaches up to 18.8, 22.8 and 27.5 MPa at 3, 7 and 28 d, respectively. Cattiite (Mg_3_(PO_4_)_2_·22H_2_O), K-struvite (KMgPO_4_·6H_2_O) and/or Na-struvite (NaMgPO_4_·6H_2_O) were identified as the main hydration products of EMPC. The development of EMPC mainly involves the dissolution of a phosphorus source, MgO and Mg_2_SiO_4_, formation of hydration product as binder, and combination of the unreacted raw materials together by binders to build a compact form.

## 1. Introduction

Electric furnace ferronickel slag (EFS) is an industrial by-product that originates from the smelting process of nickel and iron-nickel alloys. With the development of the iron-nickel industry, the annual discharge amount of ferronickel slag in China exceeds 30 million tons, which has become the fourth largest smelting slag [1]. However, the utilization rate of EFS is less than 10% [2,3,4]. Consequently, a large amount of EFS has to be disposed of in landfills and/or sites, which threatens the local ecological environment.

Currently, many investigations have been carried out on the disposal and utilization of EFS in the field of materials preparation, such as geopolymer [5,6,7], acid-base cement [8,9], concrete aggregate [10,11], cement clinker [8,12], insulation blocks [13], acoustic materials [14], inorganic mineral fibers [15,16], fertilizers [17] and metal recovery [18,19]. Particularly, the utilization of EFS for preparing magnesium cement is one of the most practicable approaches in view of its high-magnesium characteristics.

Phosphate cements, also known as chemically bonded phosphate ceramics, are usually manufactured through the reaction of acidic phosphate and basic metal oxides, forming insoluble phosphate salts as binders. According to the types of metal oxides, phosphate cements are commonly divided into zinc phosphate cement (ZPC), magnesium phosphate cement (MPC) [20,21,22], calcium phosphate cement (CPC) [23,24] and iron phosphate cement (IPC) [25]. Among these, MPC has attracted the most attention and been widely investigated, especially in terms of its thermodynamic characteristics [4,26], influencing factors and mechanical properties [27], formation mechanism [28,29] and applications [30,31]. Its popularity is due to its excellent properties, such as quick setting and high temperature resistance, which can be used as refractory materials [32], filling material for hard tissue [33] and/or concrete repair for road and airport [34]. However, the magnesium oxide used in the MPC system is normally dead-burned at a temperature of 1300–1700 °C, which gives rise to the high energy consumption and production cost. To cut down the cost and green the preparation process, the industrial by-products or solid wastes have been employed as alternative precursors and/or supplementary materials in the preparation of phosphate cement [35,36,37,38].

Herein, to further develop the industrial wastes-based MPC, the high-magnesium EFS was used as the substitute for magnesium oxide to react with three kinds of acidic components, including phosphoric acid (H_3_PO_4_, PA), sodium dihydrogen phosphate (NaH_2_PO_4_, SDP) and potassium dihydrogen phosphate (KH_2_PO_4_, PDP). The influence of the phosphorus sources (PS) and raw materials ratio on the compressive strength, early hydration temperature and microstructure of EFS-based magnesium phosphate cement (EMPC) pastes were investigated. Aiming to understand the hydration of EMPC, the hydration product and evolutions of pH and ions (Mg, K, Na, P, Si) concentrations were analyzed by X-ray diffractometry (XRD), Fourier transform infrared spectroscopy (FTIR), thermos-gravimetric and differential scanning calorimeter (TG/DTG) and inductively coupled plasma-atomic emission spectroscopy (ICP-AES).

## 2. Materials and Methods

### 2.1. Raw Materials

The EFS used in this investigation was kindly supplied by Guangxi Nickel Co., Ltd., in Guangxi, China. The EFS was dried in an oven at 80 °C for 24 h and ground for 3 h in a planetary ball mill (ND7-2L, Nanda Tianzun, Nanjing, China). The particle-size distribution and the chemical composition of EFS powders were measured by Laser diffraction particle size analyzer (master sizer 3000, Malvern Panalytical, Malvern, England) and X-ray fluorescence (XRF, 1800, Shimadzu, Kyoto, Japan) [38], respectively.

Meanwhile, according to our previous investigation, to strengthen the early hydration activity of EFS, a certain amount of pure magnesium oxide (MgO) could be added as the supplementary material in the preparation process [39].

The acidic phosphorus sources used in this study include phosphoric acid (H_3_PO_4_, PA), sodium dihydrogen phosphate (NaH_2_PO_4_, SDP) and potassium dihydrogen phosphate (KH_2_PO_4_, PDP). All the reagents were purchased from Tianjin Fengchuan Chemical Reagent Science and Technology Co., Ltd. (Tianjin, China).

### 2.2. Sample Preparation

Three series of EMPC pastes were prepared and designated as A, B and C by using PA, SDP and PDP as the phosphorus sources, respectively. For each series, the raw material mass ratio was in the range of 3.0–7.0. Meanwhile, a 5% mass ratio of MgO was added as the supplementary material for the preparation of the EMPC paste. The mix proportion of EMPC paste is exhibited in Table 1. The preparation of EMPC was performed via the following steps. First, for series B and C, the dry raw materials including EFS, MgO and SDP or PDP according to the mix proportion were dry mixed for 5 min. Next, the water was added into the mixtures at the water-to-cement mass ratio of 0.2 and wet mixed for another 5 min. After that, the fresh mixed paste was casted into cube molds with dimensions of 20 mm × 20 mm × 20 mm. Finally, the hardened pastes were demolded after 1 d and left to cure at room temperature until the pre-set testing time (3, 7 and 28 d).

In addition, in order to accurately investigate the hydration behavior, EMPC suspensions (classified as S) with different phosphorus sources were prepared according to the mix proportions listed in Table 1. The evolution of pH and ions concentrations was timely monitored. Meanwhile, the control suspension series (named as SC) without the addition of MgO was also prepared using PDP as the acid source to verify the role of EFS for the provision of Mg ion.

### 2.3. Test Methods and Characterization

For the EMPC paste, the compressive strength was determined by a compression machine (YAW-100D, Jinan Kesheng, Jinan, China) at 3, 7 and 28 d. All the batch tests were measured on three parallel samples, and the arithmetic means were determined as the final value of compressive strength (GB/T 50081–2019). The crushed samples were collected and dried for the following analysis. The microstructure of EMPC paste was observed by SEM/EDS (Quanta 200 FEG, FEI, Hillsboro, OR, USA). The chemical structure of EFS and EMPC were determined with FTIR (Bruker Ten-sor27, Karlsruhe, German), and the FTIR spectra with a resolution of 4 cm^−1^ in the range of 4000–400 cm^−1^ was collected using the KBr pellet method. The phase compositions of EFS and EMPC were analyzed by an XRD (X’ Pert-PRO, Philips, Amsterdam, Netherlands) with a Cu Kα radiation (1.5406 Å) from 10° to 80° at a scanning speed of 10°/min. The thermal stability of EMPC was explored by TG/DTG (STA 2500, Netzsch, Selb, Germany) at a heating rate of 10 °C/min in a flowing nitrogen atmosphere [25,40].

For EMPC suspension, the evolution of the pH of the EMPC suspension was measured with a pH acidity meter (PHS-3C, INESA, Shanghai, China). For ion (Mg, K, Na, P, Si)-concentration measurements, a disposable syringe filter (Zhiyu, Taixing, China) assembled with a 0.22 μm filter membrane was used to collect and filter the EMPC suspension (0.5 mL) at each of the set reaction times. Then, the obtained filtrate was diluted to 20 times and immediately acidified with enough nitric acid solution (2%, mass concentration) to prevent the precipitation reaction between Mg^2+^, K^+^, Na^+^ and PO_4_^3−^ before determining the ions concentrations using ICP-AES (Optima 3200 RL, Perkin Elmer, Waltham, MA, USA) [40].

## 3. Results and Discussion

### 3.1. Physical and Chemical Properties of EFS

**Figure 1:**
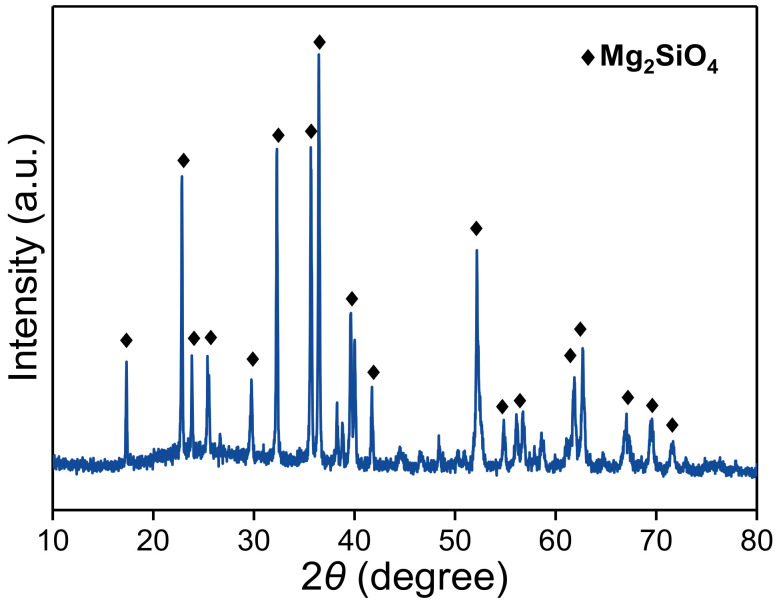
XRD pattern of EFS.

The particle-size distribution of EFS powders is shown in Table 2. The percentage of the particles with a size of less than 48.08 μm is about 91%. The chemical composition of EFS is listed in Table 3. The results showed that the EFS contains a large amount of silica, magnesia and iron oxide, accounting for 89.78% of the total composition. It is worth noting that the amount of MgO in EFS is up to 23.05%, suggesting a potential resource for the preparation of magnesium phosphate cement. The mineralogical component of EFS is shown in Figure 1. EFS mainly contains crystalline forsterite (Mg_2_SiO_4_) phase, as well as a certain amount of amorphous phase.

### 3.2. Mechanical Property and the Evolution of the Hydration Temperature of EMPC Paste

#### 3.2.1. Effect of the Raw Materials Ratio on the Compressive Strength of EMPC Paste

**Figure 2:**
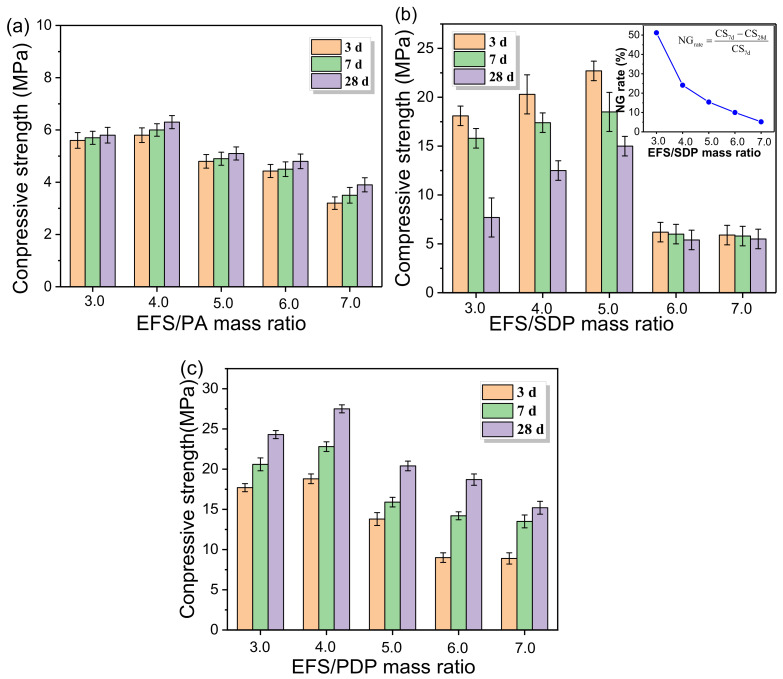
Compressive strengths of EMPC prepared with different EFS/PS ratios hydrated for 3, 7, 28 d. (**a**) EFS-H_3_PO_4_ system, (**b**) EFS-NaH_2_PO_4_ system and the negative growth rate (NG rate) of compressive strength with different EFS/SDP ratios at ages from 7 to 28 d, (**c**) EFS-KH_2_PO_4_ system.

Figure 2 presents the compressive strengths of EMPC pastes with different phosphorus sources and the raw materials ratio at 3, 7 and 28 d. As shown in Figure 2a, the compressive strength first slightly increased and then decreased with the increase in the EFS/PA ratio. When the EFS/PA ratio was 4.0, the corresponding compressive strengths of EMPC paste were up to 5.8, 6.0 and 6.3 MPa at 3, 7 and 28 d, respectively. The development of compressive strength of EMPC might be attributed to the reactions between the effective component of EFS or MgO and PA to form the hydration products, whereas the inert component of EFS acted as the aggregate in the cementitious system. When the EFS/PA was below 4.0, excess unreacted PA in the cementitious system led to a decrease in compressive strength. At a higher EFS/PA ratio, the amount of EFS apparently increased, while the formation amount of hydration products decreased due to the limited amount of PA, thus resulting in a subsequent decrease in compressive strength.

The effect of the EFS/SDP ratio on the compressive strength of EMPC pastes is shown in Figure 2b. As the EFS/SDP ratio increased, the compressive strength first increased and then decreased. When the EFS/SDP ratio was up to 5.0, the compressive strength reached up to 22.7, 18.5 and 15 MPa at 3, 7 and 28 d, respectively. Unfortunately, the compressive strength gradually decreased as the curing time increased from 3 to 28 d. This is known as the negative growth of compressive strength, which is associated with the hygroscopic cracking caused by the hygroscopicity of the surplus SDP in the system. Additionally, the negative growth rates of the compressive strength (NG rate) from 7 to 28 d were 51.2%, 24.1%, 15.4%, 10% and 5.2% at the EFS/SDP ratio of 3.0, 4.0, 5.0, 6.0, 7.0, respectively, which was in perfect accordance with the reduction in SDP in the EMPC (Table 1). The surplus SDP in the EMPC system would result in hygroscopic expansion, and the higher the amount of SDP in the EMPC, the more obvious the decline in compressive strength.

The effect of the EFS/PDP ratio on the compressive strength of EMPC pastes is shown in Figure 2c. It can be observed that the change trend of the compressive strength is similar to that of the series A. The compressive strength at 3, 7 and 28 d reached up to 18.8, 22.8 and 27.5 MPa at the EFS/PDP ratio of 4.0, respectively, which showed a significant increase in comparison to series A and B.

On the basis of the above experimental data, it can be found that the compressive strength of EMPC is significantly impacted by the phosphorus sources (PA, SDP and PDP) and EFS/PS ratio. For series A (EFS-H_3_PO_4_ system), the highest compressive strength was obtained at the EFS/PA ratio of 4.0, corresponding to 5.8, 6.0 and 6.3 MPa at 3, 7 and 28 d, respectively. Furthermore, when PDP is employed as PS, the best compressive strength reaches up to 18.8, 22.8 and 27.5 MPa at 3, 7 and 28 d, respectively, while SDP used as PS exerts varying influences on the compressive strength, and presents a negative growth as the curing age prolonged in the later hydration process.

#### 3.2.2. Early Hydration Temperature Analysis of EMPC Paste

**Figure 3:**
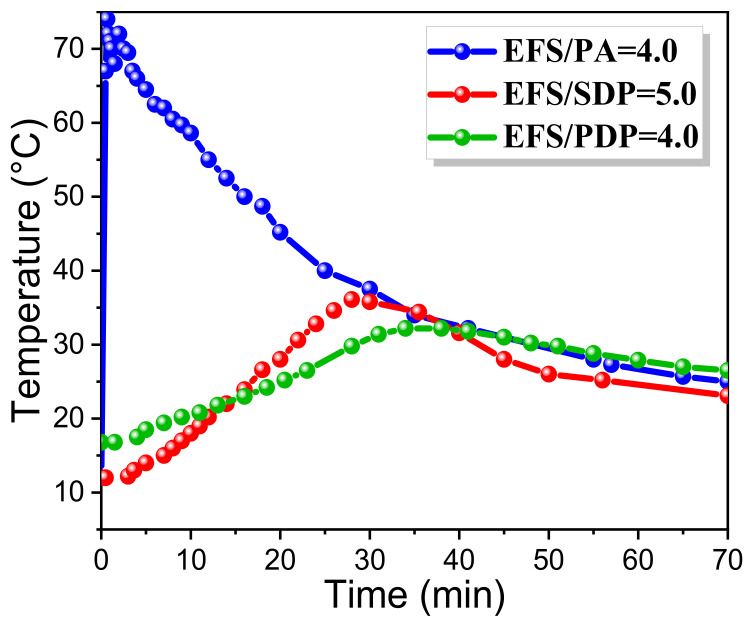
Early hydration temperature curves of EMPC with optimal proportions (A2: EFS/PA = 4.0, B3: EFS/SDP = 5.0, C2: EFS/PDP = 4.0).

The early hydration temperature of EMPC with optimal proportions (EFS/PA = 4.0, EFS/SDP = 5.0, EFS/PDP = 4.0) in the initial period (0–70 min) is shown in Figure 3. As displayed in Figure 3, the hydration of EMPC was an exothermic process, and the peaks of the hydration temperature were 74, 36, 32 °C in the EFS-H_3_PO_4_, EFS-NaH_2_PO_4_ and EFS-KH_2_PO_4_ systems, respectively. It is apparent that the temperature curve of EFS-H_3_PO_4_ system was obviously different from the others, and the hydration temperature sharply rose to 74 °C within the first minute and then slowly dropped to room temperature after hydration for 70 min. This indicates that the reaction rate between EFS and H_3_PO_4_ was extremely high, whereas a high reaction rate would lead to structural defects (such as pores and cracks) of the paste, thereby causing a reduction in the compressive strength. In comparison, the acid-base reactions in both the EFS-NaH_2_PO_4_ and EFS-KH_2_PO_4_ systems are more moderate.

### 3.3. Characterization of EMPC Pastes

#### 3.3.1. Mineralogical Composition Analysis

**Figure 4:**
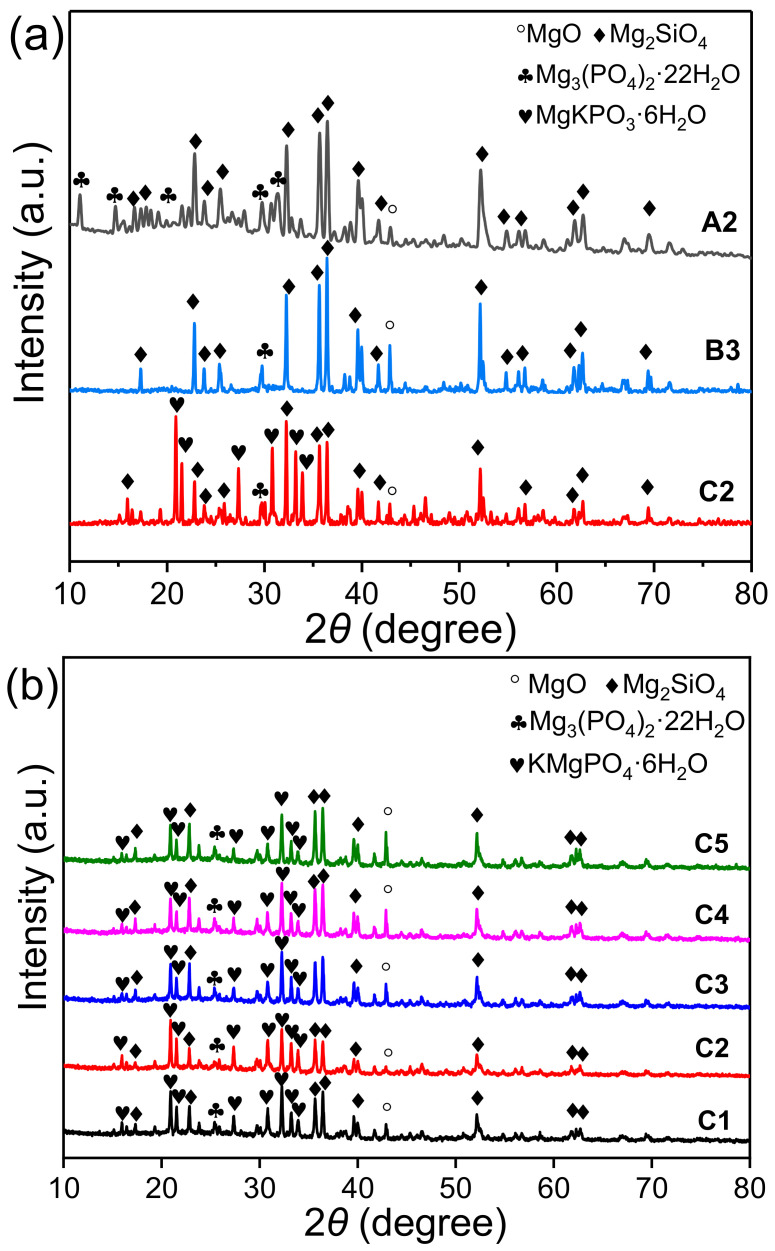
XRD patterns of EMPC pastes hydrated for 28 d: (**a**) A2: EFS/PA = 4.0, B3: EFS/SDP = 5.0 and C2: EFS/PDP = 4.0, (**b**) C1–C5: EFS/PDP ratios of 3.0, 4.0, 5.0, 6.0, 7.0.

The XRD patterns of EMPC pastes (A2: EFS/PA = 4.0, B3: EFS/SDP = 5.0 and C1–C5: EFS/PDP ratios of 3.0, 4.0, 5.0, 6.0, 7.0) at 28 d are shown in Figure 4. As shown in Figure 4a, a certain amount of magnesium phosphate (Mg_3_(PO_4_)_2_·22H_2_O) was formed as the hydration product in three EMPC systems. The intensity of Mg_3_(PO_4_)_2_·22H_2_O characteristics peaks in the A2 sample (EFS-H_3_PO_4_ system) was obviously stronger than that of sample B3 (EFS-NaH_2_PO_4_ system) and C2 (EFS-KH_2_PO_4_ system), indicating that Mg_3_PO_4_·22H_2_O was the main hydration product in series A. For series C, apart from Mg_3_PO_4_·22H_2_O, an abundant of K-struvite (KMgPO_4_·6H_2_O) was also formed, and the amount of K-struvite increased with the decrease in the EFS/PDP mass ratio (Figure 4b). It manifests that more of the hydration product of KMgPO_4_·6H_2_O was formed as the cementitious phase in the sample C2 to strengthen the mechanical strength, which was in accordance with the results in Figure 2c. However, aside from a trace of Mg_3_(PO_4_)_2_·22H_2_O, no other crystalline hydration product was formed in the sample B3. According to the information the reference reported, amorphous phase-like NaMgPO_4_·6H_2_O may be formed in series B [41]. Notably, the characteristic peaks of magnesium silicate (Mg_2_SiO_4_) and magnesium oxide (MgO) were detected for three series EMPC pastes, indicating that a part of the raw materials remained unreacted.

#### 3.3.2. SEM Analysis of EMPC Paste

**Figure 5:**
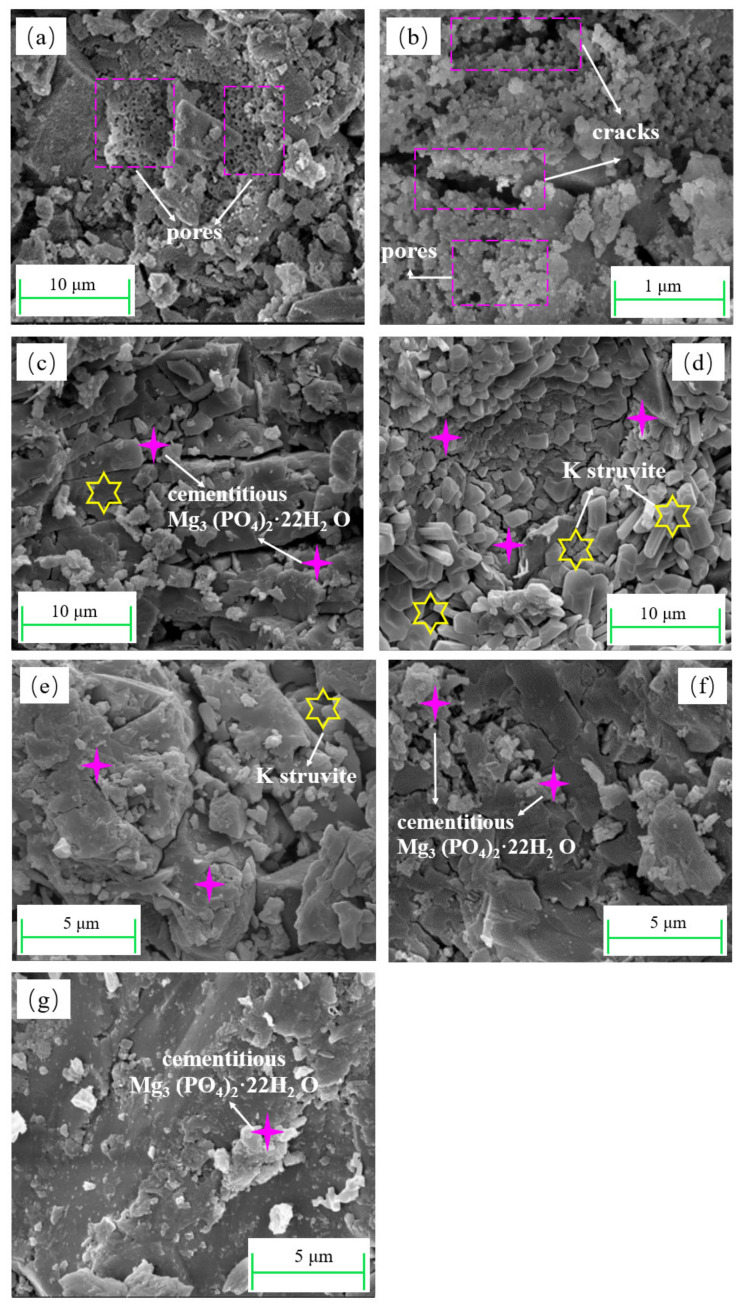
SEM images of EMPC pastes with different phosphorus sources hydrated for 28 d: (**a**) A2: EFS/PA = 4.0, (**b**) B3: EFS/SDP = 5.0, (**c**) C1: EFS/PDP = 3.0, (**d**) C2: EFS/PDP = 4.0, (**e**) C3: EFS/PDP = 5.0, (**f**) C4: EFS/PDP = 6.0, (**g**) C5: EFS/PDP = 7.0.

The influence of phosphorus sources on the microstructure of EMPC pastes is shown in Figure 5. As shown in Figure 5a, a large number of pores and cracks were presented on the fracture surface of EMPC paste (A2) with EFS/PA = 4.0 at 28 d resulting in a low compressive strength, which was in agreement with the results of Figure 3 and Figure 2a. It may be attributable to the reaction between EFS and H_3_PO_4_ releasing more heat to cause the water evaporation to further form additional defects in the EMPC paste. In the EFS-NaH_2_PO_4_ system (Figure 5b, EFS/SDP = 5.0, B3), it can also be observed that a lot of cracks presented in the EMPC paste, which may provide the opportunity for the air and water to access the inside of the paste, causing the moisture adsorption of unreacted SDP, thereby leading to the decrease in compressive strength. The SEM images of the EFS-KH_2_PO_4_ systems (EFS/PDP = 4.0, 5.0, 6.0, 7.0) are shown in Figure 5c–g. The compactness and hydration products of pastes strongly depended on the EFS/PDP ratio. As the EFS/PDP ratio increased, the proportion of the binder and aggregate decreased. At EFS/PDP = 4.0, a number of rod-like crystals were formed, suggested to be the K-struvite by the EDS analysis. Thus, the hydrated EMPC paste prepared with PDP seemed compact and cohesive, contributing to the high compressive strength (Figure 2c).

#### 3.3.3. FTIR Analysis

**Figure 6:**
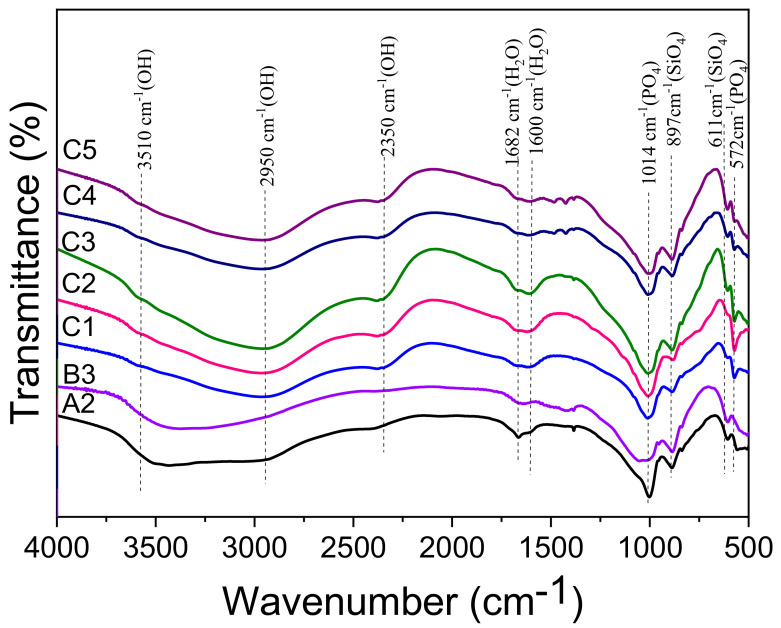
FTIR spectra of A2, B3 and series C (C1–C5) pastes hydrated for 28 d.

Figure 6 displays the FTIR spectra of A2, B3 and C1–C5 pastes hydrated for 28 d. With regard to Mg_3_(PO_4_)_2_·22H_2_O and KMgPO_4_·6H_2_O or NaMgPO_4_·6H_2_O, the absorbance bands at 1014 and 572 cm^−1^ were assigned to the PO_4_^3–^ antisymmetric stretching and the P-O bending vibrations, respectively [42,43]. The bands at 897 and 611 cm^−1^ were assigned to SiO_4_ stretching (800–1100 cm^−^^1^) and bending (650–500 cm^−1^) vibrations, respectively. The band located at 1682–1600 cm^−1^ was attributed to the bending vibrations of the H_2_O molecules. The broad band in the 3800–2200 cm^−1^ region was assigned to the O-H stretching vibration. According to the references [40,44,45,46], the broad band at 3510 cm^−1^ in the sample A2 and B3 was assigned to the OH stretching vibration of the crystalline water, whereas it shifted to the low frequency (2950, 2350 cm^−1^) with the existence of fairly strong hydrogen bonds in the structure of KMgPO_4_·6H_2_O or NaMgPO_4_·6H_2_O.

#### 3.3.4. TG/DTG Analysis

**Figure 7:**
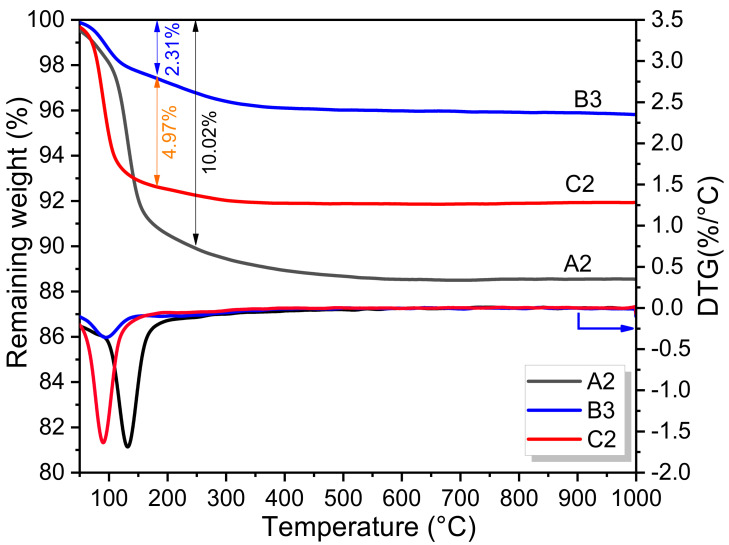
TG/DTG curves of sample A2, B3 and C2.

Figure 7 shows the TG/DTG curves of sample A2, B3 and C2 hydrated for 28 d. It is apparent that the weight-loss of EMPC pastes with different phosphorus sources between 90 and 130 °C presented a great gap. The largest weight loss was as high as 10.02% in the sample A2, attributed to the thermal decomposition of Mg_3_PO_4_·22H_2_O. The weight loss related to the decomposition of MgKPO_4_·6H_2_O in the sample C2 was up to 7.28%. Although the bound water in the Mg_3_PO_4_·22H_2_O (70.3%) was higher than that of MgKPO_4_·6H_2_O (40.6%), the weight-loss rate was obviously inferior to the theoretical ratio of 1.73. It suggests that more MgKPO_4_·6H_2_O was formed in sample C2, which was in agreement with the XRD results, as given in Figure 4. In the sample B3, the weight loss at around 90 °C was as low as 2.31%, indicating that less of the hydration product was formed. The similar decomposition curves in both sample B3 and C2, suggested that amorphous NaMgPO_4_·6H_2_O may be responsible for the weight loss in the EFS-NaH_2_PO_4_ system [40].

### 3.4. Evolutions of pH and Ions Concentrations in EMPC Suspension

**Figure 8:**
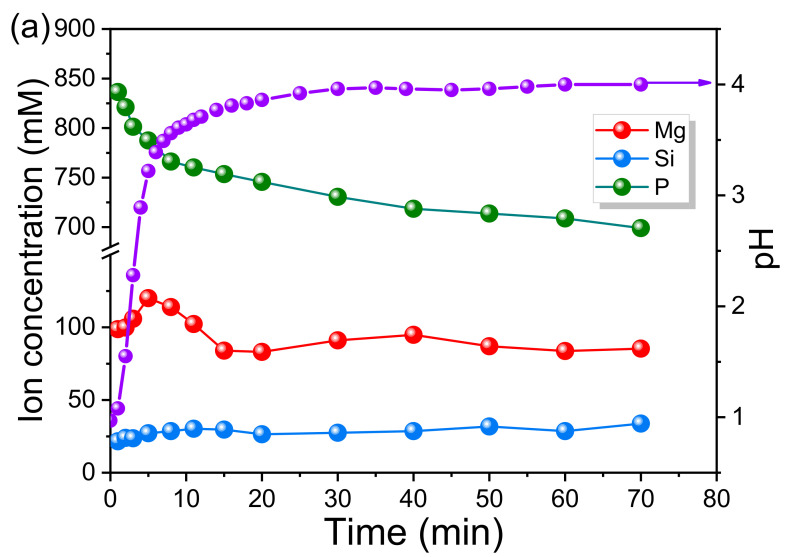

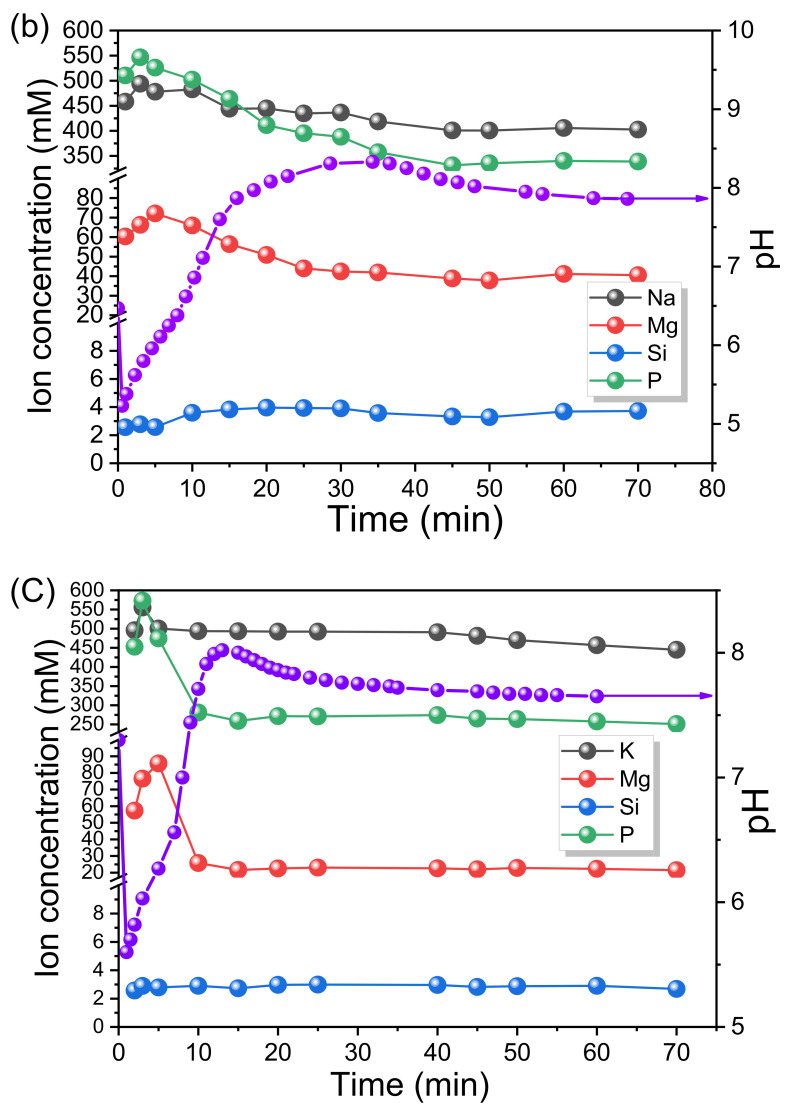

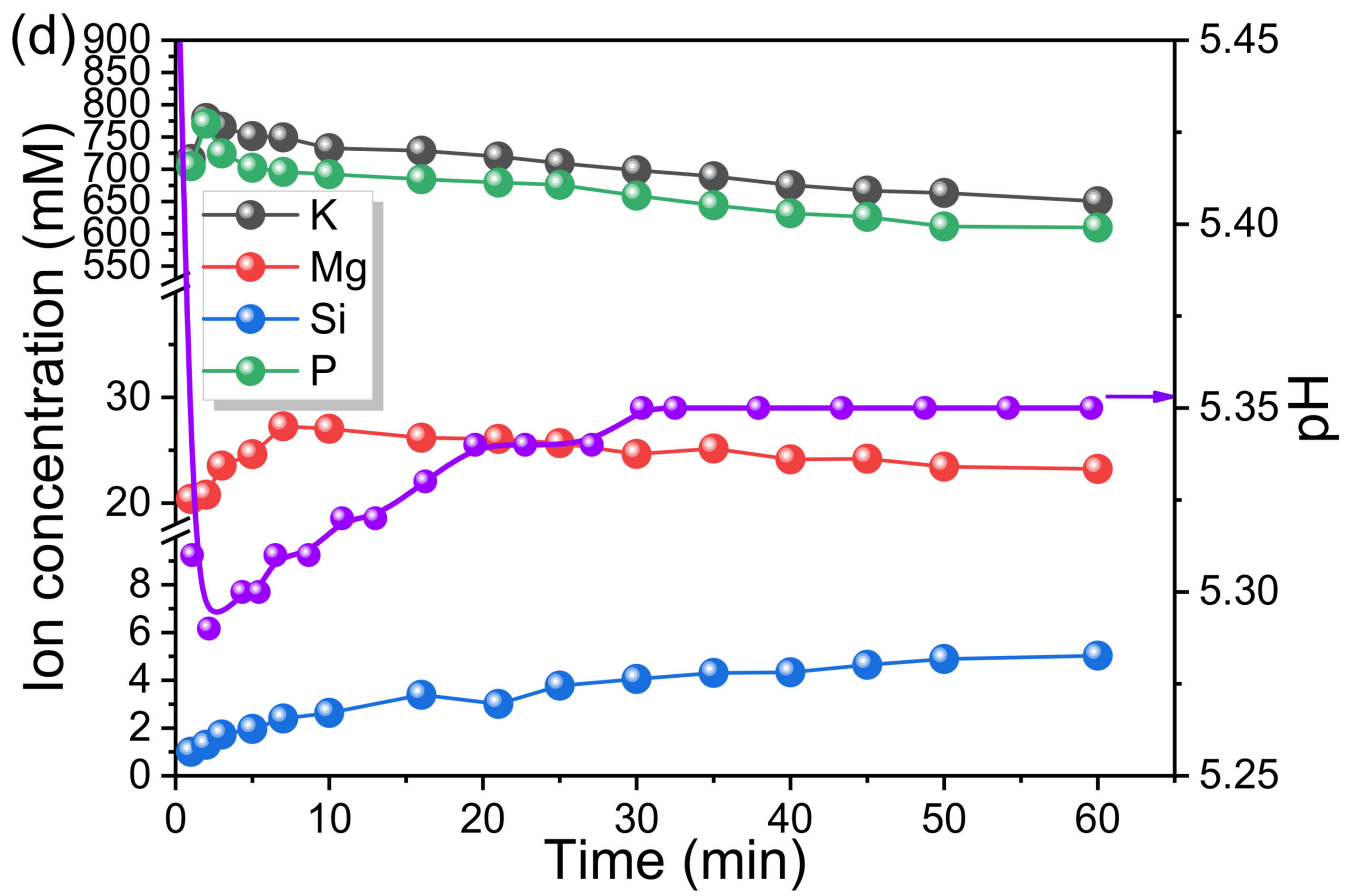
The evolution of ions concentration and pH value in the EMPC suspensions: (**a**) SA2: EFS/PA = 4.0; (**b**) SB2: EFS/SDP = 4.0; (**c**) SC2: EFS/PDP = 4.0; (**d**) SCC2: EFS/PDP = 4.0 without MgO.

To further reveal the hydration pathway of EMPC, the changes of pH and ions (i.e., Mg, P, Si, Na and K) concentrations in the EMPC suspensions (SA2, SB2, SC2 and control group of SCC2) were investigated, and the results are shown in Figure 8.

As shown in Figure 8a, the pH value of SA2 sharply increased at the initial stage and then slowly increased to a final steady state at around 4.0 (70 min). While the evolutions for Si, P and Mg concentrations were quite different, the Si concentrations continued to rise throughout, confirming the continual dissolution of Mg_2_(SiO_4_) from EFS. For the P and Mg concentrations, at the initial stage (within 5 min), they presented an opposite trend, suggesting only few amount of hydrates were formed. Additionally, the release rate of Mg from MgO and/or EFS was faster than the consummation rate of Mg, which reflected an overall increase in Mg and a decrease in P concentrations. After 5 min, the Mg concentration started to decrease quickly, indicating the fast precipitation of hydrates, until it became constant after 30 min. As displayed by Figure 4, the hydration product Mg_3_PO_4_·22H_2_O was formed in sample A2. As shown in Figure 8b,c, a similar trend was observed in both SB2 and SC2. Apart from the Si concentration, all the ions concentrations including Na (or K), P, Mg first increased and then decreased. At the first stage (within 5 min), the increase in concentrations was attributed to the dissolution of SDP, PDP, MgO and EFS. According to the change of pH, it can be inferred that the SDP and PDP was dissolved firstly, resulting a sharp decrease in pH (pH as low as 5.2 and 5.6, respectively). Then, the increase in pH occurred due to the dissolution of MgO and EFS reacting with H^+^ (pH as high as 8.0 and 8.3, respectively). Between 5 and 20 min, the apparent changes in Na (or K), P and Mg concentrations were attributed to the main precipitation reaction of hydrates, which formed crystalline KMgPO_4_·6H_2_O or amorphous NaMgPO_4_·6H_2_O [47]. However, it is worth noting that the variation degree of Mg, P and K in SC2 (59.87, 303.19, 108.7 mM) was higher than that of SB2 (28.14, 210.1, 84.47 mM), indicating that more precipitates were formed in SC2. In addition, the result of the control experiment for SCC2 without MgO is shown in Figure 8d. It can be observed that a certain amount of Mg was released from EFS, which indicated that the hydrates KMgPO_4_·6H_2_O could be also formed through the acid-base reaction between EFS and KH_2_PO_4_.

### 3.5. Discussion of the Hydration Mechanism of EMPC

**Figure 9:**
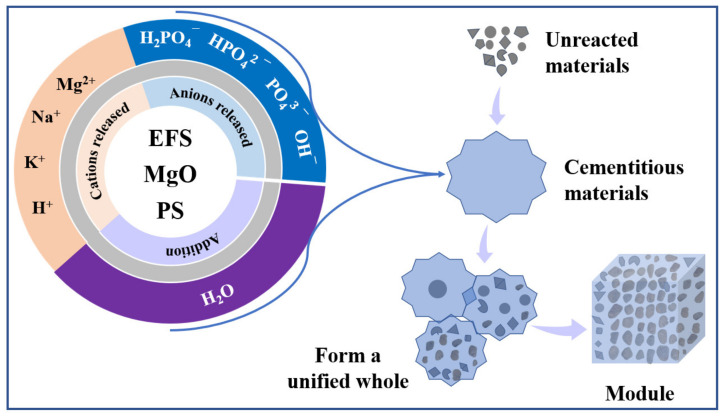
Scheme of formation mechanism of the EMPC.

On the basis of the above mentioned experimental and characterization results, the hydration pathway of EMPC can be concluded via the following three steps. The formation mechanism of the EMPC paste is schematically described in Figure 9.

(1) The dissolution of acidic phosphorus sources (H_3_PO_4_, NaH_2_PO_4_, KH_2_PO_4_) releases the H^+^, H_2_PO_4_^−^, HPO_4_^2−^, PO_4_^3−^, Na^+^ or K^+^, which can be described as Equations (1)–(6).

(1) $$H_{3}{\mathrm{PO}}_{4}⇌H_{2}{\mathrm{PO}}_{4}{}^{-}+H^{+}$$

(2) $$H_{2}{\mathrm{PO}}_{4}{}^{-}⇌{\;\mathrm{HPO}}_{4}{}^{2-}+H^{+}$$

(3) $${\mathrm{HPO}}_{4}{}^{2-}⇌{\mathrm{PO}}_{4}{}^{3-}+H^{+}$$

(4) $${\mathrm{MeH}}_{2}{\mathrm{PO}}_{4}\to H_{2}{\mathrm{PO}}_{4}{}^{-}+{\mathrm{Me}}^{+}(\mathrm{Me}=\mathrm{Na}\;\mathrm{or}\;K)$$

(5) $$H_{2}{\mathrm{PO}}_{4}{}^{-}⇌{\;\mathrm{HPO}}_{4}{}^{2-}+H^{+}$$

(6) $${\mathrm{HPO}}_{4}{}^{2-}⇌{\mathrm{PO}}_{4}{}^{3-}+H^{+}$$

(2) MgO or Mg_2_SiO_4_ contained in EFS dissolves in the above-acidic solution (pH = 1.0, 5.2, 5.6 for EFS-H_3_PO_4_, EFS-NaH_2_PO_4_, EFS-KH_2_PO_4_ system, respectively) to release the Mg^2+^ (Equations (7) and (8)). The dissolution rate of Mg_2_SiO_4_ is slower than MgO, leading that the decrease of Mg is in different degree with the later acid-base reaction [39].

(7) $$\mathrm{MgO}+H^{+}\to {\mathrm{Mg}}^{2+}+{\mathrm{OH}}^{-}$$

(8) $${\mathrm{Mg}}_{2}{\mathrm{SiO}}_{4}+4H^{+}\to 2{\mathrm{Mg}}^{2+}+{\mathrm{Si}(\mathrm{OH})}_{4}$$

(3) The precipitation reaction occurs between the acid phosphate and pre-released cations (Equations (9) and (10)), for which Mg_3_PO_4_·22H_2_O, KMgPO_4_·6H_2_O, NaMgPO_4_·6H_2_O are formed principally as binders to secure the unreacted Mg_2_SiO_4_ or other materials contained in the EFS, and finally build a hardened paste with a high compressive strength [48].

(9) $$3{\mathrm{Mg}}^{2+}+2H_{2}{\mathrm{PO}}_{4}{}^{-}+4{\mathrm{OH}}^{-}+18H_{2}O\to {\mathrm{Mg}}_{3}{{(\mathrm{PO}}_{4})}_{2}\cdot 22H_{2}O$$

(10) $${\mathrm{Mg}}^{2+}{+K}^{+}{+H}_{2}{\mathrm{PO}}_{4}{}^{-}+6H_{2}O\to {\mathrm{KMgPO}}_{4}\cdot {6H}_{2}O$$

## 4. Conclusions

The preparation of magnesium phosphate cement using high-magnesium EFS is one of the most practicable approaches. In this study, three species of phosphorus sources were adopted as the acidic component to react with the EFS when preparing EFS-based magnesium phosphate cement (EMPC). The effect of raw materials’ mass ratio on the mechanical strength, early hydration temperature and microstructure of EMPC paste were systematically investigated. The highest compressive strength of EMPC paste is 6.3, 22.7 and 27.5 MPa when hydrated for 28 d at the EFS/PA = 4.0, EFS/SDP = 5.0 and EFS/PDP = 4.0, respectively. The XRD, FTIR and TG/DTG results verified the development of EMPC mainly due to the formation of hydration products, i.e., Mg_3_PO_4_·22H_2_O, KMgPO_4_·6H_2_O, NaMgPO_4_·6H_2_O as the binders to connect the unreacted EFS contributing to a high compressive strength. However, the hydration of EMPC is a multi-stage process, including the dissolution of PS, MgO and Mg_2_SiO_4_, and precipitation reaction when forming K/Na-struvite and Mg_3_PO_4_·22H_2_O binders.

## Figures and Tables

**Table 1:** Mix proportion of EMPC paste and EMPC suspension.

Series	PS	No.	EFS/PS ^a^	EFS (g)	PS (g)	MgO (g)	W/C ^b^
A	H_3_PO_4_	A1	3.0	90.0	30.0	4.5	0.2
		A2	4.0	96.0	24.0	4.8	0.2
		A3	5.0	100.0	20.0	4.5	0.2
		A4	6.0	102.9	17.1	5.1	0.2
		A5	7.0	105.0	15.0	5.3	0.2
B	NaH_2_PO_4_	B1	3.0	90.0	30.0	4.5	0.2
		B2	4.0	96.0	24.0	4.8	0.2
		B3	5.0	100.0	20.0	4.5	0.2
		B4	6.0	102.9	17.1	5.1	0.2
		B5	7.0	105.0	15.0	5.3	0.2
C	KH_2_PO_4_	C1	3.0	90.0	30.0	4.5	0.2
		C2	4.0	96.0	24.0	4.8	0.2
		C3	5.0	100.0	20.0	4.5	0.2
		C4	6.0	102.9	17.1	5.1	0.2
		C5	7.0	105.0	15.0	5.3	0.2
S	H_3_PO_4_	SA2	4.0	40.0	10.0	2.0	2.0
	NaH_2_PO_4_	SB2	4.0	40.0	10.0	2.0	2.0
	KH_2_PO_4_	SC2	4.0	40.0	10.0	2.0	2.0
SC	KH_2_PO_4_	SCC2	4.0	40.0	10.0	/	2.0

Notations: ^a^ mass ratio; ^b^ includes EFS, PS and MgO.

**Table 2:** Particle size distribution of EFS powders (wt.%).

Particle Size (μm)	Percentage (wt.%)
<6.30	24.10
6.30~12.70	28.52
12.7~20.69	16.90
20.69~48.08	21.56
48.08~69.68	5.00
>69.68	3.92

**Table 3:** Chemical composition of EFS (wt.%).

Compound	Percentage (wt.%)
SiO_2_	55.18
MgO	23.05
Fe_2_O_3_	11.55
Al_2_O_3_	3.37
CaO	1.85
MnO	1.79
SO_3_	0.37
K_2_O	0.05
Others	2.79

## Data Availability

Not applicable.
